# Anti-biofilm properties of laser-synthesized, ultrapure silver–gold-alloy nanoparticles against *Staphylococcus aureus*

**DOI:** 10.1038/s41598-024-53782-x

**Published:** 2024-02-10

**Authors:** Nils Heine, Katharina Doll-Nikutta, Frederic Stein, Jurij Jakobi, Alexandra Ingendoh-Tsakmakidis, Christoph Rehbock, Andreas Winkel, Stephan Barcikowski, Meike Stiesch

**Affiliations:** 1 Department of Prosthetic Dentistry and Biomedical Materials Science, Hannover Medical School, Carl-Neuberg-Straße 1, 30625 Hannover, Germany; 2 Lower Saxony Centre of Biomedical Engineering, Implant Research and Development, Stadtfelddamm 34, 30625 Hannover, Germany; 3 Technical Chemistry I, University of Duisburg Essen, Universitaetsstr. 7, 45141 Essen, Germany

**Keywords:** Microbiology, Medical research, Nanoscience and technology

## Abstract

*Staphylococcus aureus* biofilm-associated infections are a common complication in modern medicine. Due to inherent resilience of biofilms to antibiotics and the rising number of antibiotic-resistant bacterial strains, new treatment options are required. For this purpose, ultrapure, spherical silver–gold-alloy nanoparticles with homogenous elemental distribution were synthesized by laser ablation in liquids and analyzed for their antibacterial activity on different stages of *S. aureus* biofilm formation as well as for different viability parameters. First, the effect of nanoparticles against planktonic bacteria was tested with metabolic activity measurements. Next, nanoparticles were incubated with differently matured *S. aureus* biofilms, which were then analyzed by metabolic activity measurements and three dimensional live/dead fluorescent staining to determine biofilm volume and membrane integrity. It could be shown that AgAu NPs exhibit antibacterial properties against planktonic bacteria but also against early-stage and even mature biofilms, with a complete diffusion through the biofilm matrix. Furthermore, AgAu NPs primarily targeted metabolic activity, to a smaller extend membrane integrity, but not the biofilm volume. Additional molecular analyses using qRT-PCR confirmed the influence on different metabolic pathways, like glycolysis, stress response and biofilm formation. As this shows clear similarities to the mechanism of pure silver ions, the results strengthen silver ions to be the major antibacterial agent of the synthesized nanoparticles. In summary, the results of this study provide initial evidence of promising anti-biofilm characteristics of silver–gold-alloy nanoparticles and support the importance of further translation-oriented analyses in the future.

## Introduction

Bacterial implant-associated infections frequently cause severe complications in modern medicine^1–4^. The treatment of these infections remains difficult although a plethora of antibiotics is available, since infection sites are difficult to reach for systemically administered antibiotics due to missing vascularization of implants^5–7^, the rise of multidrug-resistant bacterial strains^8,9^, and the formation of bacterial biofilms^10–12^. Biofilms are single- or multi-species microbial communities encapsulated in a matrix of self-produced extracellular polymeric substances and inherently resistant to antimicrobial substances^11,13,14^. One of the most relevant bacterial species in implant-associated infections is the gram-positive *Staphylococcus aureus*. It has already been detected on orthopedic, cardiovascular, and dental implants^15^. It is also one of the most relevant species with regard to antibiotic resistance due to the SCC*mec* gene complex that encodes resistance to most β-lactam antibiotics^16^. One example is methicillin-resistant *S. aureus* (MRSA) with an estimated prevalence of almost 150,000 cases and over 7,000 deaths per year in the countries of the European Union and the European Economic Area^17^. In southern Europe, 24–43% of isolated *S. aureus* strains from invasive infections in 2021 were identified as MRSA^18^.

To overcome resistance against antibiotics, alternative treatments are within the focus of current research. For this purpose, several approaches, like modification of antimicrobials to circumvent resistance mechanisms, compounds that inhibit mechanisms of resistance, new antimicrobial target proteins, inhibiting virulence factors, nanoparticles, antimicrobial peptides, phages, or antisense oligonucleotides are pursued^19^. Amongst them, silver has already frequently been studied for medical applications^20^. Silver ions prevent growth of bacteria by inhibiting several enzymes relevant for glycolysis, the pentose phosphate pathway, the electron transport chain, and the oxidative stress defense system^21^. Due to their large effective surface area and consequent high ion release, nanoparticles are an effective way to apply silver and have already been used for medical applications, e.g., wound and burn treatment^22^. However, silver ions have also shown toxicity towards eukaryotic cells^23^. Once inside the cell, silver ions can induce production of reactive oxygen species (ROS), which lead to protein denaturation, DNA damage, and finally cell death^24^. The relation of cytotoxicity to antibacterial activity differs between studies. A study by Greulich et al*.*^25^ reports silver ions and chemically synthesized silver nanoparticles with an average size of 70 nm to be toxic towards bacteria and human cells in the same concentration range. Jena et al*.*^26^ found their chemically synthesized chitosan-coated silver nanoparticles to be antibacterial towards human pathogens without harming macrophages at a certain concentration. In case of a study investigating silver nanoparticles produced by laser ablation with an average size of ≤ 10 nm, bacteria and human cells reacted to similar concentrations, narrowing the therapeutic window—defined as a concentration range that is antibacterial but not cytotoxic^27^. In another study, the impact of Ag NPs and silver nitrate on gene expression in ecological communities was studied and the silver ions were identified as the main toxic species here as well^28^.

To reduce cytotoxicity, silver–gold-alloy nanoparticles (AgAu NPs) have been introduced^29–31^. It has already been shown that alloying silver nanoparticles with gold reduces toxicity towards human cell cultures disproportionally to the silver-to-gold ratio^27^. This is hypothesized to be due to a reduced silver ion release caused by the gold^31^. Gold can also serve as an anchor to selectively couple target-specific entities to nanoparticles, which would be interesting for further modifications^32^. When AgAu NPs are synthesized by laser ablation in liquid (LAL), ultrapure, spherical solid solution alloy nanoparticles with homogenous elemental distribution defined by the target, reproducible particle sizes, and free of additional surfactants can be produced^33–35^. Due to the complete miscibility of silver and gold, all silver-to-gold ratios can be achieved^33^.

The antibacterial effect against *S. aureus* of AgAu NPs synthesized by LAL has already been tested in an agar diffusion assay^27^. The MIC was 25 µg/mL for AgAu NPs consisting of 80% silver atoms and 20% gold atoms and stabilized by BSA. However, in this assay, planktonic bacteria are streaked out on agar plates containing AgAu NPs and grow in colonies^27^. Therefore, the physiological biofilm morphology that is mostly the cause for implant-associated infections and considerably more tolerant against treatment is not considered^10,36^. To the authors’ knowledge, there is no record in the literature how AgAu NPs synthesized by LAL would affect *S. aureus* biofilms.

This study aims to analyze the effect of surfactant free AgAu NPs synthesized by LAL on different stages of *S. aureus* biofilm formation and on different parameters for bacterial viability. For this purpose, they were first tested against planktonic bacteria with metabolic activity measurements. They were then incubated with differently matured *S. aureus* biofilms. The biofilms were analyzed by metabolic activity measurements and three dimensional live/dead fluorescent staining to determine biofilm volume and membrane integrity. To further investigate the antibacterial mechanism of AgAu NPs, gene expression of *S. aureus* after treatment with AgAu NPs was analyzed and compared to the effect of pure silver ions.

## Results

### Generation of AgAu NPs with monodisperse size distribution and molar gold fraction of 0.2

**Figure 1:**
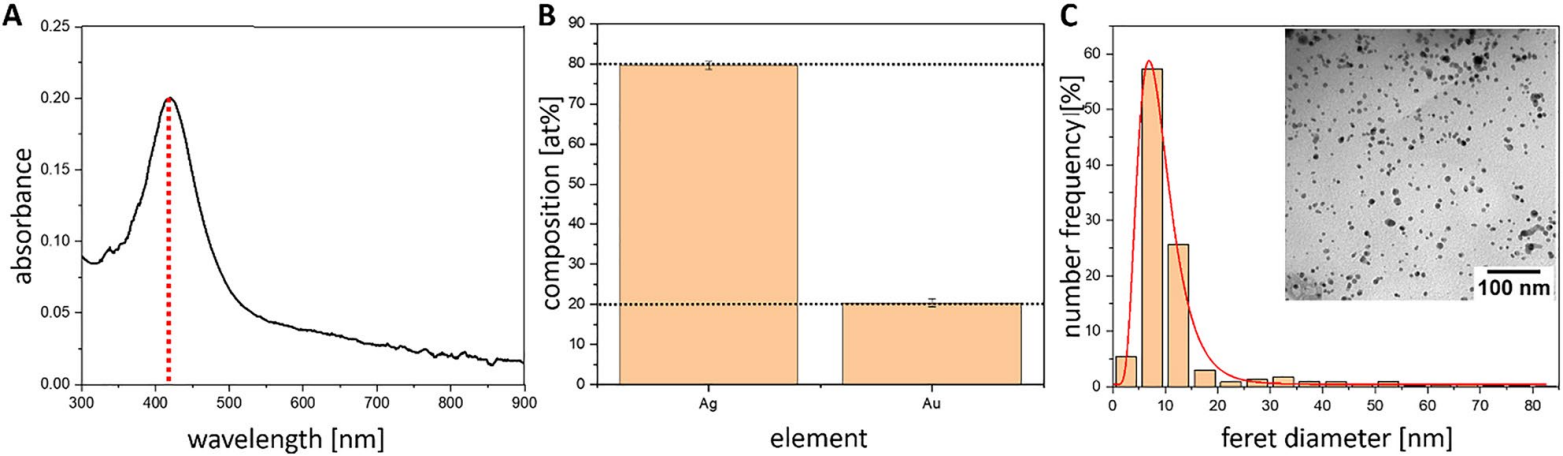
(**A**) Exemplary UV–Vis spectrum of AgAu nanoparticles. The dotted line indicates the maximum of the localized SPR peak maximum at ~ 415 nm. (**B**) Bulk composition of representative AgAu nanoparticles determined by XRF. Error bars are standard deviations from 3 measurements, the dotted lines indicate the nominal compositions (Ag,Au) of the bulk targets. (**C**) Number-weighted particle size distribution obtained from TEM as well as an exemplary TEM image (insert). Size distributions were fitted with a log-normal distribution function and are based on N = 1,000 individual particles.

To verify whether the desired elemental composition was achieved and to analyze the size distribution of nanoparticles, UV–Vis spectroscopy, X-ray-fluorescence (XRF) spectroscopy, and transmission electron microscopy (TEM) were used. UV–Vis spectroscopy (Fig. 1A) revealed a single absorbance peak at roughly 415 nm. A gold molar fraction of 0.2 determined by XRF can be observed in Fig. 1B. Number-weighted particle size distributions derived from TEM analysis showed a mean nanoparticle diameter of 8 ± 3.5 nm (Fig. 1C). Please note that the AgAu NPs used here have been thoroughly characterized in the literature. For example high-resolution TEM images and EDX single particle analysis can be found in the study by Neumeister et al*.*^34^, crystallographic analyses was done by Prymak et al*.*^35^, and release kinetics on a single particle level were reported by Al-Zubeidi et al*.*^37^. Furthermore, a detailed characterization of the particles´ surface oxidation and surface chemistry can be found in Stein et al*.*^38^*.*

### Reduced metabolic activity of planktonic *S. aureus* upon treatment with AgAu NPs

**Figure 2:**
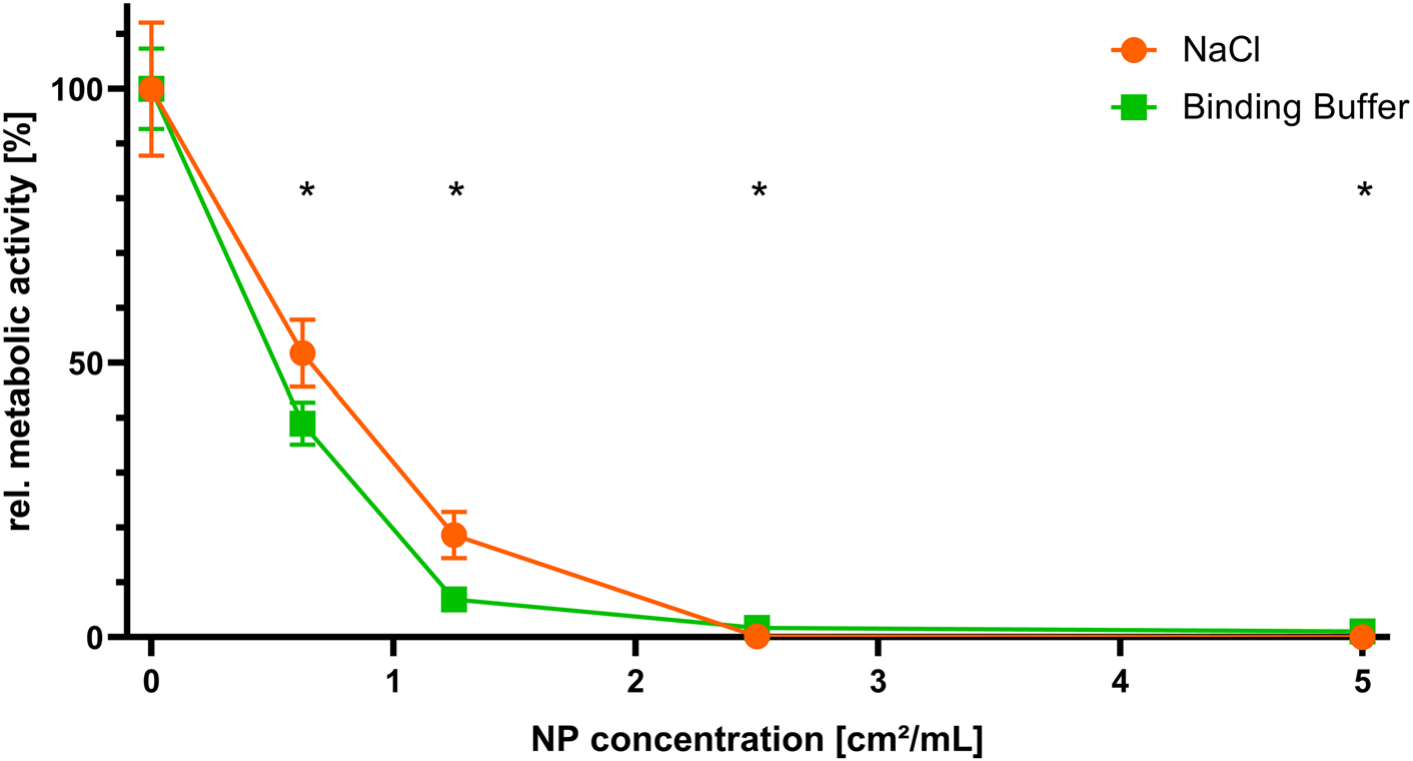
Mean ± standard deviation of relative metabolic activity after incubation of planktonic *S. aureus* with different concentrations of nanoparticles (NP) consisting of 80 mol% silver and 20 mol% gold for 3 h in two different media. Statistically significant differences between concentrations for the same medium with *p* ≤ 0.05 determined by an ordinary one-way ANOVA with Tukey’s test for multiple comparisons can be found in Supplementary Tables 1 (for NaCl) and 2 (for binding buffer). Statistically significant differences between media for the same concentration with *p* ≤ 0.05 determined by an unpaired t test are depicted by (*).

First, the effect of AgAu NPs on planktonic *S. aureus* was analyzed. Viability was measured using a resazurin assay after incubation with different surface concentrations of AgAu NPs for 3 h in either 0.2 mM NaCl or binding buffer. The results are shown in Fig. 2. All data was normalized to the untreated group (0 cm^2^/mL) in the respective medium. Metabolic activity of planktonic bacteria decreased with increasing concentration of AgAu NPs to a comparable degree in both media. A surface concentration of 1.25 cm^2^/mL was sufficient to decrease relative metabolic activity statistically significantly by about 81 ± 4% in 0.2 mM NaCl and 93 ± 2% in binding buffer. This difference between media was small but also statistically significant.

### Different effects of AgAu NPs on *S. aureus* biofilm viability, volume and membrane integrity

Mature biofilms are known to be much more resistant to antimicrobial substances than planktonic bacteria^11,36^. To take this into account, *S. aureus* was incubated in tryptone soya broth supplemented with 10% yeast extract (TSBy) and 50 mM glucose at 37 °C for either 5 h or 24 h to obtain biofilms of different maturity. These biofilms were subsequently treated with AgAu NPs for 18 h in either 0.2 mM NaCl or binding buffer. Like planktonic bacteria, biofilms were analyzed with a resazurin assay to determine their metabolic activity and data were normalized to the control (0 cm^2^/mL). To additionally analyze volume and membrane integrity, biofilms were fluorescently stained with live/dead fluorochromes and images were taken using confocal laser scanning microscopy (CLSM).

**Figure 3:**
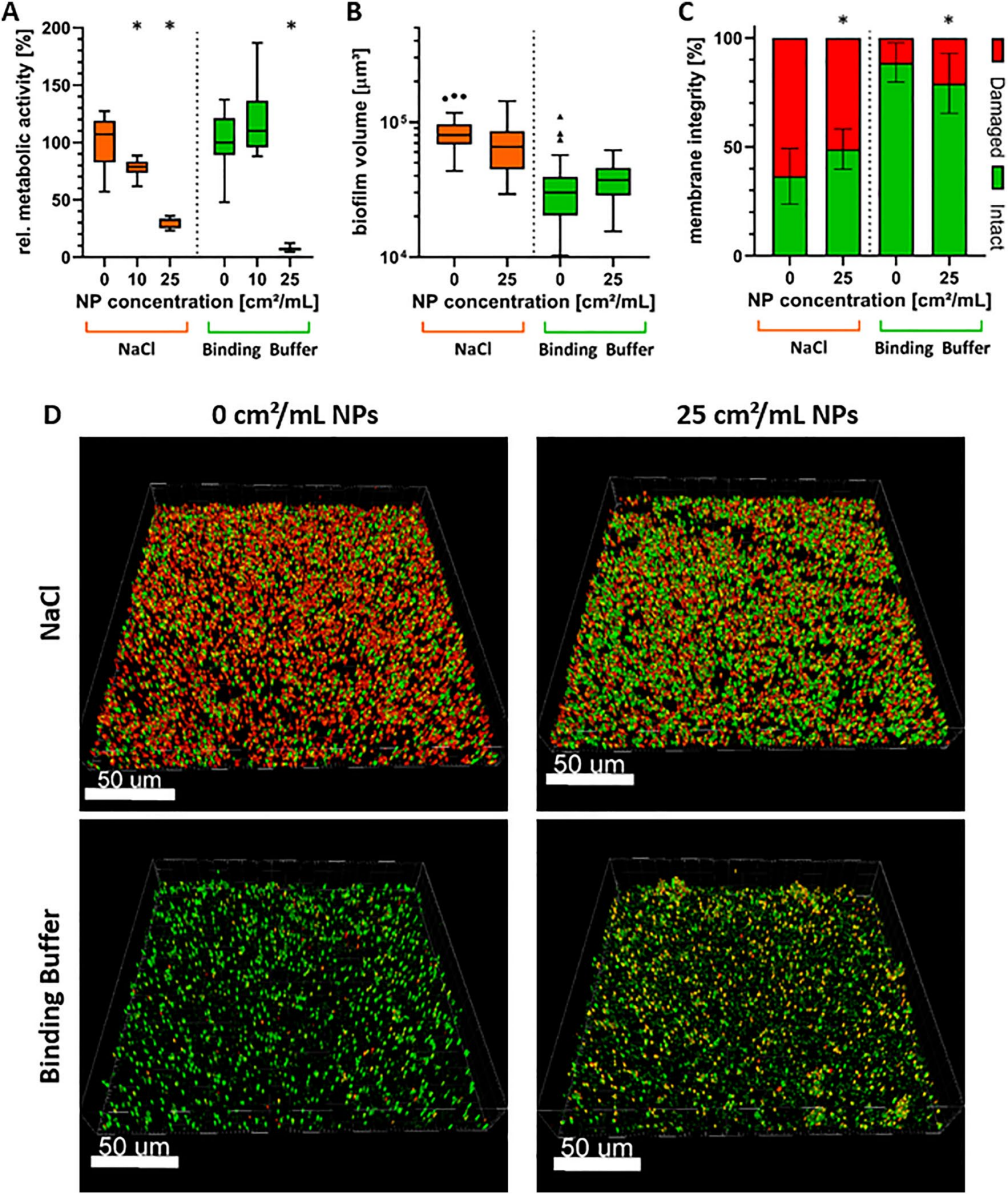
Effect of nanoparticles consisting of 80 mol% silver and 20 mol% gold on 5 h old *S. aureus* biofilms after incubation for 18 h in two different media. (**A**) Tukey box plots of biofilm metabolic activity determined by resazurin assay. (**B**) Tukey boxplots of biofilm volume and (**C**) mean ± standard deviation of intact/damaged membrane distribution analyzed by live/dead fluorescence staining, CLSM imaging and evaluation with BitPlane Imaris. Statistically significant differences with *p* ≤ 0.05 to the control (0 cm^2^/ml) are indicated by (*) and were determined by (**A**) one-way ANOVA with Dunnett’s test for multiple comparisons, (**B**) Friedman’s test with Dunn’s test for multiple comparisons, and (**C**) Kruskal–Wallis’ test with Dunn’s test for multiple comparisons. (**D**) Representative 3D-reconstructed CLSM images of live/dead fluorescence stained, untreated and treated biofilms. Cells with intact membrane (live) are depicted in green and cells with damaged membrane (dead) are depicted in orange/red.

The results for the 5 h old biofilms are shown in Fig. 3. Metabolic activity (Fig. 3A) was reduced with increasing AgAu NP concentration in both media with a statistically significant decrease by 70 ± 5% in NaCl and 93 ± 2% in binding buffer when using 25 cm^2^/mL. For NaCl, the bacterial viability was already statistically significantly reduced by 22 ± 8% at 10 cm^2^/mL. The difference between 10 cm^2^/mL and 25 cm^2^/mL in NaCl was also statistically significant. In case of binding buffer, no difference could be detected for the lower concentration. Based on these results, a concentration of 25 cm^2^/mL was chosen for microscopic biofilm evaluation. As shown in Fig. 3B,D, biofilm volume did not change upon treatment with AgAu NPs. On average it reached 7.8 × 10^4^ ± 2.7 × 10^4^ µm^3^ and 3.5 × 10^4^ ± 1.6 × 10^4^ µm^3^ in NaCl and binding buffer, respectively. In contrast, differences could be observed for biofilm membrane integrity (Fig. 3C,D). For binding buffer, untreated biofilms had 11 ± 9% cells with damaged membranes and their amount statistically significantly increased when incubated with AgAu NPs to 21 ± 14%. Untreated biofilms in NaCl showed 63 ± 13% cells with damaged membranes. The amount statistically significantly decreased upon treatment with AgAu NPs to 51 ± 9%. For both, biofilm volume and membrane integrity, the differences between media were statistically significant as well.

**Figure 4:**
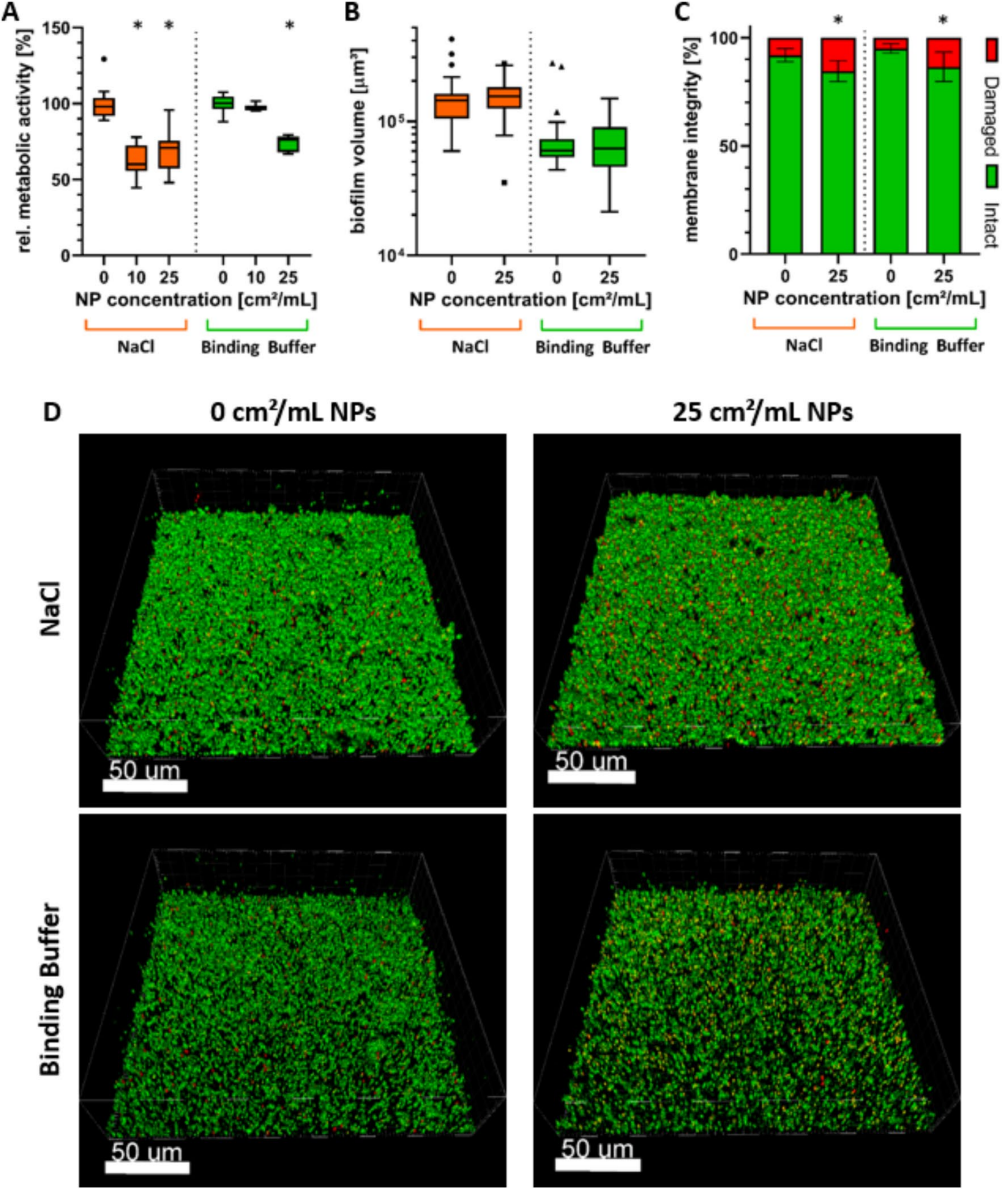
Effect of nanoparticles consisting of 80 mol% silver and 20 mol% gold on 24 h old *S. aureus* biofilms after incubation for 18 h in two different media. (**A**) Tukey box plots of biofilm metabolic activity determined by resazurin assay. (**B**) Tukey boxplots of biofilm volume and (**C**) mean ± standard deviation of intact/damaged membrane distribution analyzed by live/dead fluorescence staining, CLSM imaging and evaluation with BitPlane Imaris. Statistically significant differences with *p* ≤ 0.05 to the control (0 cm^2^/ml) are indicated by (*) and were determined by (**A**) Kruskal–Wallis’ test with Dunn’s test for multiple comparisons, (**B**) Friedman’s test with Dunn’s test for multiple comparisons, and (**C**) one-way ANOVA with Sidak’s test for multiple comparisons. (**D**) Representative 3D-reconstructed CLSM images of live/dead fluorescence stained, untreated and treated biofilms. Cells with intact membrane (live) are depicted in green and cells with damaged membrane (dead) are depicted in orange/red.

In Fig. 4, the results for the 24 h old biofilms are depicted. Metabolic activity (Fig. 4A) was statistically significantly reduced by 31 ± 14% in NaCl and by 26 ± 5% in binding buffer when using 25 cm^2^/mL of AgAu NPs. In NaCl, the reduction was statistically significant with 37 ± 11% at 10 cm^2^/ml. There was no statistically significant difference between the two treatment concentrations in NaCl. In binding buffer, treatment with the lower concentration did not result in a statistically significant difference in metabolic activity to the control. Again, a concentration of 25 cm^2^/mL was chosen for microscopic biofilm evaluation. Biofilm volume (Fig. 4B) was unaffected by AgAu NPs and was on average 1.5 × 10^5^ ± 5.6 × 10^4^ µm^3^ and 7.2 × 10^4^ ± 3.8 × 10^4^ µm^3^ in NaCl and binding buffer, respectively. Membrane integrity (Fig. 4C,D) however, was statistically significantly reduced in both media. Untreated biofilms consisted of 8 ± 3% and 5 ± 2% cells with damaged membranes in NaCl and binding buffer, respectively, increasing to 15 ± 5% and 13 ± 7% in the presence of the AgAu NPs. The differences between media were statistically significant for biofilm volume and the untreated group of membrane integrity.

### Complete diffusion of AgAu NPs through *S. aureus* biofilms

**Figure 5:**
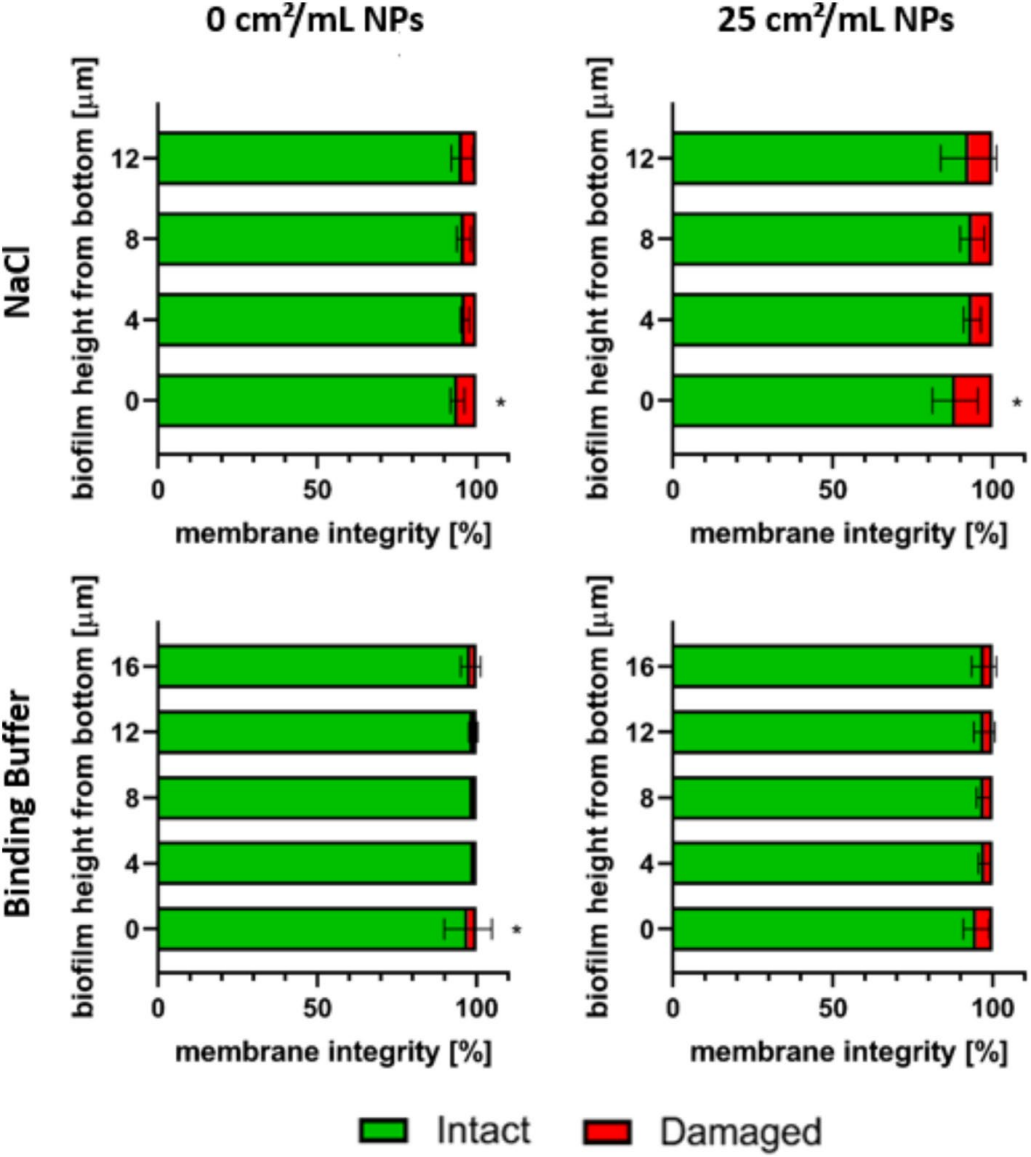
Mean ± standard deviation of intact/damaged membrane distribution throughout different z-planes in *S. aureus* biofilms after 24 h of cultivation followed by 18 h of incubation with or without nanoparticles consisting of 80 mol% silver and 20 mol% gold in two different media. (*) indicates a statistically significant difference with *p* ≤ 0.05 of the bottom plane to all other planes in the respective biofilm, determined by Kruskal–Wallis’ test with Dunn’s test for multiple comparisons.

One of the major defense mechanisms of biofilms is the EPS matrix serving as a diffusion barrier for antimicrobial substances^36^. To analyze whether AgAu NPs diffuse through 24 h old *S. aureus* biofilms, single planes were extracted from the 3D images created by CLSM every 4 µm starting from the bottom of the biofilms. In these planes, membrane integrity was determined. The results are shown in Fig. 5. It can be observed that in all biofilms the highest percentage of damaged cells was in the bottom layer. For untreated biofilms, the percentage of bacteria with damaged membranes was 6 ± 2% and 3 ± 7% in NaCl and binding buffer, respectively. For treated biofilms, the percentage of damaged cells is increased evenly in the whole biofilm and the bottom layer still contains the highest amount with 12 ± 7% in NaCl and 5 ± 4% in binding buffer.

### AgAu NPs target expression of *S. aureus* metabolic genes and increase ROS activity

**Figure 6:**
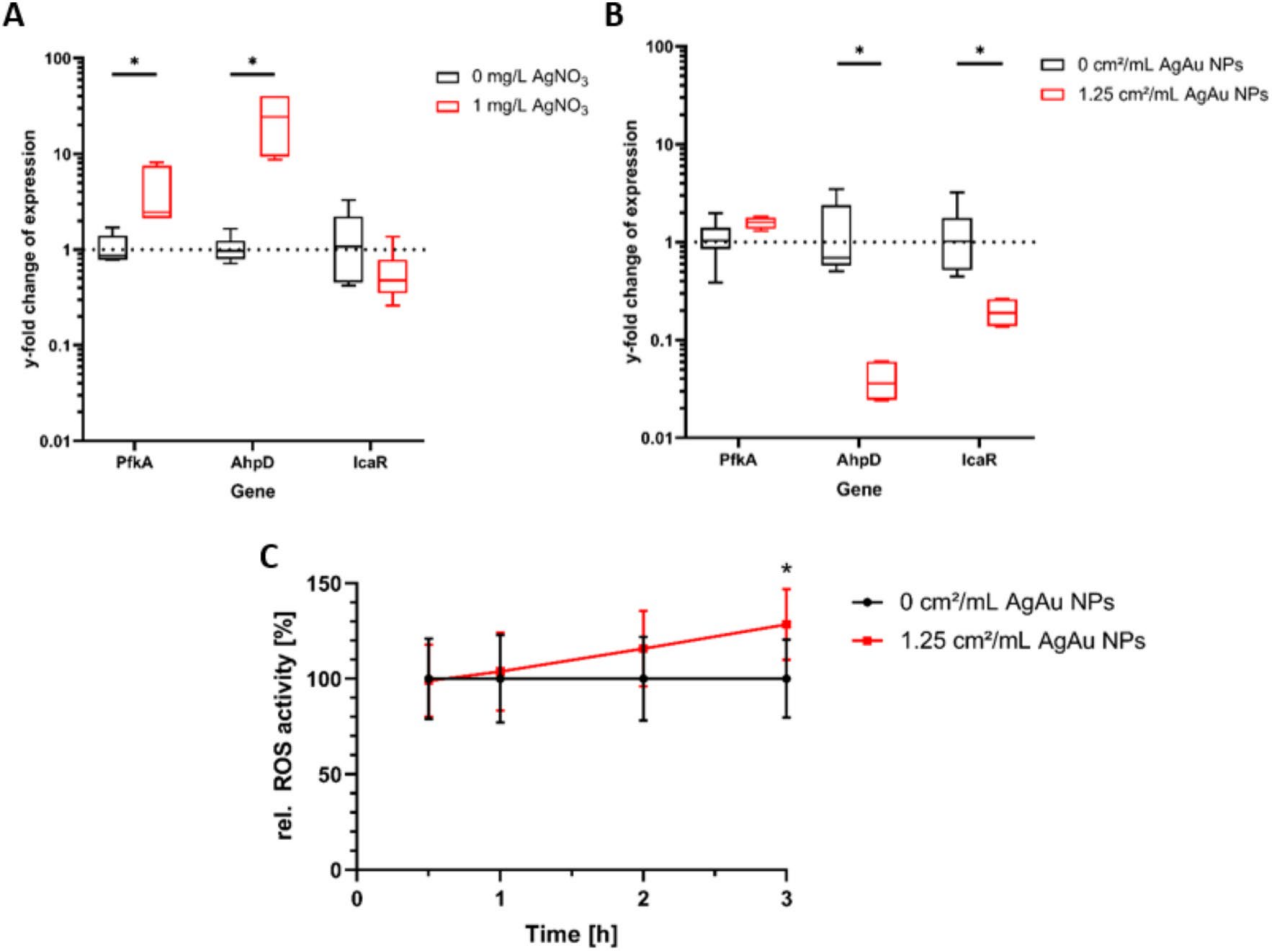
Effect of AgNO_3_ (**A**) and nanoparticles consisting of 80 mol% silver and 20 mol% gold (**B**) on gene expression of planktonic *S. aureus* after incubation of 3 h in binding buffer. Results were normalized to the *rrsA* housekeeping gene. (**C**) Mean ± standard deviation of relative total reactive oxygen species (ROS) activity over time in *S. aureus* incubated with 1.25 cm^2^/mL of nanoparticles consisting of 80 mol% silver and 20 mol% gold in binding buffer normalized to the untreated control. Statistically significant differences with *p* ≤ 0.05 between treated and untreated groups are indicated by (*) and were determined by unpaired t tests.

To further analyze the antibacterial mechanism of AgAu NPs, expression of known silver target as well as biofilm relevant genes was quantified in planktonic *S. aureus* after treatment with 1 mg/L AgNO_3_ or 1.25 cm^2^/mL AgAu NPs using qRT-PCR and compared to a control (0 mg/L or 0 cm^2^/mL, respectively). The data was analyzed using the 2^−ΔΔ*C*^_T_-method^39^ with *rrsA* as housekeeping gene. The results are shown in Fig. 6A,B as y-fold expression compared to the housekeeping gene and the control.

In Fig. 6A, the influence of silver ions from dissolved AgNO_3_ can be observed. Expression of *pfkA* and *ahpD* is statistically significantly increased to 4.4 ± 2.9-fold and 24.6 ± 16.5-fold, respectively. Expression of *icaR* is slightly reduced to 0.6 ± 0.4, however, this reduction is not statistically significant. The influence of AgAu NPs is depicted in Fig. 6B. Here, the expression of *pfkA* is also increased. With an increase to 1.6 ± 0.2-fold, it is less pronounced than for AgNO_3_ and with a p-value of 0.0668 not defined as statistically significant. However, it is close to the threshold of 0.05. In contrast to AgNO_3_, the expression of *ahpD* is statistically significantly reduced to 0.04 ± 0.02-fold under the influence of AgAu NPs. The expression of *icaR* is statistically significantly reduced to 0.2 ± 0.06-fold like for AgNO_3_.

Additionally, as reactive oxygen species (ROS) are considered to be a major factor of the antibacterial activity of silver, the total intracellular ROS activity of planktonic *S. aureus* treated with AgAu NPs was measured and compared to an untreated control. In Fig. 6C, it can be observed that the total ROS activity of *S. aureus* treated with AgAu NPs compared to cells without treatment increased over the course of 3 h. 30 min after starting treatment, average ROS activity was 1% lower than the control, but it increased over time and reached a statistically significant 28% higher activity after 3 h.

## Discussion

AgAu NPs are a promising approach as alternative antibacterial substance due to the combination of antibacterial and biocompatible elements. Synthesis by LAL allows for ultrapure, spherical AgAu NPs with homogenous elemental distribution and reproducible particle sizes. However, so far, the antibacterial effect of these particles has not been tested on physiologically relevant biofilms, which are known for their inherent resistance to antimicrobial agents. In this study, the effect on *S. aureus* biofilms, one of the major pathogens in implant-associated infections, was investigated. Albeit biofilms are considerably more tolerant than planktonic bacteria, an antibacterial effect throughout the entire thickness of the biofilms was observed. The AgAu NPs showed their strongest effect on metabolic activity, which could be additionally verified by targets on the molecular level.

Initial characterization of the AgAu NPs generated by LAL in this study showed a clear absorbance peak in UV–Vis-extinction spectroscopy associated to the localized surface plasmon resonance (SPR) of a colloid consisting of 80 mol% silver and 20 mol% gold. As only one peak is observable, large deviations in composition as well as formation of larger fractions of pure Ag and Au nanoparticles could be excluded. Previous studies found the elemental distribution within these nanoparticles to be homogenous^34,35^. A more detailed recent analysis by XPS revealed that there is a slight enrichment of gold in the near-surface volume for the sample containing 80 mol% silver and 20 mol% gold used in this study^38^. The SPR maximum at ~ 415 nm is in good accordance with previous findings and can be associated with a gold molar fraction of ~ 0.2, which is close to the nominal composition of the target^34,35,38^. The results are further confirmed by XRF analysis, where a gold molar fraction of 0.2 was determined for the bulk colloid which is in line with previous characterization approaches based on XRD^35^ as well as single particle TEM-EDX^34^. Number-weighted particle size distributions derived from TEM analysis indicate a monodisperse and monomodal particle size distribution with a mean diameter of 8 ± 3.5 nm, with errors derived from the variance (width) of the log-normal distribution curves. Therefore, it can be concluded that the nanoparticle colloids used in this study had a monodisperse particle size distribution and a gold molar fraction of 0.2, closely matching that of the target used during ablation. In a previous study, the nanoparticles were found to be single crystals with a face centered cubic (fcc) structure^35^. Al-Zubeidi et al*.*^37^ analyzed the silver release kinetics of AgAu NPs on a single particle level. They found that alloying with gold reduced the silver leaching rate and identified two different leaching stages. The first one being dependent on silver content, while the second one depended on the oxidation potential of the particles and diffusion of silver ions through the lattice. The second leaching phase is slower and occurs once the relative gold content in the lattice reaches a certain point.

The elemental distribution of 80 mol% silver and 20 mol% gold was chosen for the synergistic properties of both metals. While pure silver results in the highest antibacterial activity of nanoparticles, only alloying it with gold allowed for cytocompatibility^27,40^. However, adding gold to the nanoparticles also changes the magnitude of the antibacterial effect. Grade et al*.*^27^ could show that by introducing 20 mol% gold, the minimum inhibitory concentration (MIC) of the nanoparticles doubled. By using nanoparticles consisting of 50 mol% silver and 50 mol% gold, the MIC increased by 800%. This is hypothesized to be due to a reduced silver ion release caused by the gold^31^. For the treatment of biofilms, it can be assumed that a much higher dose of nanoparticles is required than for planktonic bacteria. With that in mind, the 80 mol% silver and 20 mol% gold distribution was chosen for its stronger antibacterial effect.

When these particles were used for antibacterial testing in this study, all assays were done in 0.2 mM NaCl and in binding buffer. NaCl was the solution in which the nanoparticles were synthesized and in which they were most stable, based on the influence of ionic strength on stability of ligand-free particles^31^. However, 0.2 mM NaCl can be considered a hypoosmolaric system for bacteria. In contrast, binding buffer is much more comparable to physiological buffers like phosphate buffered saline based on its ion concentration. Furthermore, it enables functionality of possible further target specific entities, like species-specific aptamers^33,41^. It also has to be mentioned that nanoparticle concentrations were measured in surface area per volume instead of mass per volume in this study. This was due to the fact that silver nanoparticles do not dissolve in aqueous liquids. Instead, a small amount of silver ions is released from the particle surface and is responsible for biological effects. Hence, particle mass, which is not located at the surface, is not relevant for functionality of these particles, only the active surface contributes silver ions. This is the reason for different effects between nanoparticles and bulk materials and results in surface area being a more meaningful parameter for nanoparticle functionality than total mass^33,42^. Furthermore, surface weighted concentrations are more meaningful than mass weighted ones when comparing different nanoparticle types that may vary in particle size and density.

To initially test antibacterial activity, planktonic *S. aureus* was incubated with different concentrations of AgAu NPs for 3 h and metabolic activity was determined using a resazurin assay. Resazurin (7-hydroxy-10-oxidophenoxazin-10-ium-3-one) is a membrane permeable, blue, non-fluorescent dye. Once inside the cell, it can be irreversibly reduced by the reducing equivalents FMNH_2_, FADH_2_, NADH, or NADPH to the pink, fluorescent resorufin^43^. By measuring the increasing fluorescent signal, metabolic activity can be quantified. This assay is a well-established method to analyze antibacterial effects^44^. This initial test was done as a basis for later biofilm experiments and for comparability to other studies quantifying antibacterial activity. As metabolic activity assays are more reliable for biofilms than CFU^44^, this method was chosen for planktonic bacteria for comparability. The *S. aureus* type strain DSM 20231 was used for its reproducibility and comparability, as it has also been used in other studies researching AgAu NPs^27,45^. During all antibacterial testing, untreated bacteria were used as controls. Dead controls were not included, as, to the authors’ knowledge, there is no standard substance available that could kill mature biofilms with a similar mode of action.

The results showed that an AgAu NP concentration of 1.25 cm^2^/mL was sufficient to reduce metabolic activity by 81% to 93%, depending on the medium, which could be considered a strong antibacterial effect. Silver ions are known to affect, amongst others, the enzymes PdhB, which is responsible for a reduction of NAD^+^ to NADH during glycolysis, and 6PGDH, which is part of the reduction of NADP^+^ to NADPH/H^+^ in the pentose phosphate pathway^21^. Consequently, less of these molecules are left to reduce resazurin after treatment with AgAu NPs. Other enzymes relevant for these intracellular mechanisms are affected by silver ions as well, for example PfkA in glycolysis and Pgl in the pentose phosphate pathway^21^. This further decreases the amount of available NADH and NADPH and finally disrupts the cellular metabolism. The slight difference between binding buffer and 0.2 mM NaCl might be explained by the absolute fluorescence readout values of the resazurin assay (not shown). In 0.2 mM NaCl, the values were roughly 33% lower than in binding buffer, which means metabolic activity is generally reduced in this medium. This could most probably be attributed to the fact that 0.2 mM is a very low concentration of NaCl and the pH of the solution was between 4.5 and 5. These conditions deviate to a high extent from physiological growth conditions of bacteria. Consequently, this could mask or impair the effect of AgAu NPs by influencing the uptake of silver ions or how much metabolic activity could be further reduced by an antibacterial effect. It is also conceivable that the dissolution equilibrium of the AgAu NPs was affected by pH and particularly the chloride concentration. A strong antibacterial activity on planktonic bacteria is typical for other types of nanoparticles containing silver as well. Salunke et al. found that their biologically synthesized Ag NPs, Au NPs, and AgAu NPs exhibited a minimum inhibitory concentration (MIC) of 8 µg/mL against *S. aureus*, each^46^. Gurunathan et al. also synthesized their Ag NPs biologically and reported an MIC of 0.75 µg/mL against *S. aureus* (colony forming units (CFU) reduced by roughly 99%)^47^. Bhatia and Banerjee reported an MIC of 10 µg/mL against *S. aureus* for their chemically synthesized AgAu NPs^48^. For the AgAu NPs used in this study, with an average size of 8 nm and a molar silver-to-gold ratio of 4:1, the effective concentration of 1.25 cm^2^/mL AgAu NPs corresponds to roughly 1.75 µg/mL of silver. This is in the same magnitude as the MICs in literature, with a relatively strong antibacterial effect. The slight differences in MICs can most probably be attributed to the nanoparticle size, as it was on average 35 nm, 5 nm, and 40 nm in their studies, respectively^46–48^. Since silver from nanoparticles does not dissolve completely but rather nanoparticles release only a small amount of their silver as ions, size and, thus, active surface area affect how many nanoparticles are required for the same effect.

Compared to planktonic bacteria, biofilms are much more resistant to antibacterial action^11,13,14^. Therefore, antibacterial results cannot be simply transferred but need to be tested individually. For this purpose, AgAu NPs were first tested on early-stage biofilms, which were grown for 5 h only. Preliminary experiments had shown that, for a similarly distinct effect compared to planktonic bacteria, increasing nanoparticle concentration alone had not been sufficient but also longer treatment times were needed. Therefore, 5 h old biofilms were incubated with 10 cm^2^/mL or 25 cm^2^/mL AgAu NPs for 18 h and metabolic activity was determined using the resazurin assay. Additionally, biofilms incubated with or without 25 cm^2^/mL AgAu NPs were stained with two fluorescent dyes. Red propidium iodide can only stain cells with damaged membranes whereas green SYTO9 is able to enter and stain cells with intact membranes. CLSM was then used to acquire 3D images which could be analyzed for biofilm volume and distribution of membrane integrity. During treatment, agitation was provided with a plate shaker to ensure distribution of nanoparticles. In this study, we focused on situations comparable to inserted orthopedic implants in synovial fluid. There, no considerable flow, but a comparable gentle liquid movement is present.

The results showed an effect of AgAu NPs on metabolic activity and membrane integrity of *S. aureus* early-stage biofilms. Although a 20-times higher nanoparticle concentration was required for a similar effect than on planktonic bacteria, metabolic activity of early-stage biofilms was drastically reduced by AgAu NPs. A considerably higher resilience towards antimicrobial substances is typical for biofilms, while the here reported decrease in susceptibility is at the low end of the 10–1,000-fold decrease found in literature^11,36^. This resilience is most likely explained by reduced diffusion of the antibacterial substance through the biofilm matrix^49,50^ and a large amount of bacteria being in the stationary phase and thus being affected less by anti-metabolic action^51,52^. Another aspect, why a higher concentration of nanoparticles is needed might be the larger number of bacteria being present. The effect of AgAu NPs on membrane integrity was considerably weaker than on metabolic activity. This difference between different viability parameters can most probably be explained by silver’s mechanism of action. As described above, silver damages various metabolic pathways in *S. aureus*, strongly affecting metabolic activity^21^. However, disruption of membrane to the point of the membrane being permeable for propidium iodide seems to be a minor silver target compared to metabolic pathways. Even though other studies also reported disruption of membranes of *S. aureus* and *E. coli* by silver, they did not compare it to metabolic activity^53–55^. Therefore, future studies should address this observation in more detail by also analyzing whether reduction of membrane integrity is due to direct damage from AgAu NPs or a second-level result of cell death from damage to metabolic pathways. In contrast to metabolic activity and membrane integrity, biofilm volume was not affected by AgAu NP treatment, suggesting no eradicative properties. There are studies reporting eradicative properties for silver^56,57^. Most probably, whether eradication occurs depends on the properties of the applied silver, e.g. concentration, size, elemental composition, ligands, or other supporting substances. However, the detailed correlation to eradication still needs to be researched.

For all viability parameters of early-stage biofilms treated with AgAu NPs, there are also significant differences between the media used. In binding buffer, biofilms present with less than half of the volume compared to NaCl. This can most probably be explained by the polysorbate-type nonionic surfactant Tween 20 being an ingredient of binding buffer. As surfactant, it increases the potential of the solution to remove entities from solid surfaces. This could possibly have reduced the amount of adherent bacteria. Furthermore, membrane integrity is significantly reduced in NaCl compared to binding buffer. This is most likely due to the low pH and low osmolality of 0.2 mM NaCl being non-physiological to the bacteria. Finally, the smaller effect of AgAu NPs on metabolic activity of early-stage biofilms in NaCl can be explained likewise as for planktonic bacteria, with less reduction possible when the metabolic activity is already low. This might be enhanced by the fact that, due to biofilm volume in NaCl being higher, the ratio of nanoparticle/bacterial cell is lower. Nevertheless, even with differences in magnitude, AgAu NPs were able to exert anti-biofilm properties in both media analyzed. 0.2 mM NaCl is the ideal medium for AgAu NP stability and therefore favorable for effectiveness^31^. However, it might distort results by being non-physiological for bacteria. Binding buffer is closer to physiological conditions. Since application in 0.2 mM NaCl may not be feasible, binding buffer could be more relevant for possible future applications. Additionally, it would be especially relevant if target specific entities, like species-specific aptamers, were to be attached to the nanoparticles^33,41^.

After analyzing early-stage biofilms, the antibacterial effect of AgAu NPs was assessed for 24 h-old, mature biofilms using similar methods. As for early-stage biofilms, an effect of AgAu NPs on metabolic activity and membrane integrity could be observed for mature biofilms, while biofilm volume was unaffected. These results demonstrate that AgAu NPs can significantly damage even mature biofilms. However, the effect on metabolic activity was considerably weaker than against early-stage biofilms. This can be explained by the increased number of bacteria and thicker biofilm matrix, which roughly doubled in both media compared to the previous experiment and increase the natural biofilm resilience. The effect on membrane integrity was also reduced, but only slightly. However, membrane integrity is still affected significantly less than metabolic activity. This again strengthens the hypothesis of silver’s mechanism of action primarily targeting metabolic pathways^21^. As for early-stage biofilms, the different media influenced the magnitude of AgAu NPs’ antibacterial effect, however, differences were less pronounced. This is most likely due to the mature biofilm being more resilient towards the unphysiological properties of 0.2 mM NaCl, while the influence of the surfactant Tween 20 remains similar.

The results presented above clearly point towards a strong impact of AgAu NPs on bacterial metabolic activity in biofilms. As it has been described that silver nanoparticles affect bacteria by releasing silver ions, which are then able to enter and damage the cells^24^, this effect would be in line with the known molecular mechanism of silver ions^21^. In the following, this hypothesis was addressed in more detail.

The first precondition for AgAu NPs to affect the metabolic activity of bacteria in biofilms would be a sufficient diffusion through the biofilm matrix, which is known to act as a diffusion barrier^49,50^. To investigate this, CLSM images of mature biofilms were sliced into 4 µm thick sections horizontally by digital image analysis and then separately analyzed for membrane integrity. The results not only showed an increase of impaired membranes in the deeper layers of the biofilm independently of the medium, but also a homogeneous increase in all sections upon treatment. This indicates that AgAu NPs were able to diffuse through the entire biofilm and could damage cell membranes—and most probably also metabolic activity—even at the bottom biofilm layer. Whether the AgAu NPs themselves are able to diffuse through the matrix, or only the silver ions they release, needs to be further investigated.

Next, the influence of AgAu NPs on the expression of some bacterial genes was analyzed by RNA isolation and qRT-PCR. The influence of silver ions was also analyzed to be able to compare these two agents to get first insights into the nanoparticle’s mechanism. Two chosen genes are known silver targets: *pfkA* and *ahpD* encode phosphofructokinase A of glycolysis and alkylhydroperoxidase D in the oxidative stress response system, respectively^21^. The third selected gene, *icaR,* represses the *ica* operon and thus decreases biofilm formation, which would be interesting regarding future applications^58^. Additionally, the effect of AgAu NPs on total ROS activity was analyzed.

Treatment with AgAu NPs resulted in a slightly increased expression of *pfkA* and a decreased expression of *ahpD* and *icaR* to 0.04-fold and 0.2-fold, respectively. Silver ion treatment was applied in form of AgNO_3_ and resulted in a fourfold and 25-fold increased expression of *pfkA* and *ahpD,* respectively, and a slightly decreased expression of *icaR*. By analyses on RNA- and protein-level, Wang et al. could demonstrate that upregulation of a gene was indirectly caused by the inhibition of the encoded enzyme by silver ions^21^. For downregulation, a likewise promoting effect on the protein level can be assumed, for example by damage to relevant feedback loops. Upregulation of *pfkA* by AgAu NPs is in line with the results for pure silver ions and also with those from Wang et al.^21^. This points towards a damage of glycolysis also by AgAu NPs and/or its released silver ions, which would additionally fit to the reduced metabolic activity values. As glycolysis is an important virulence factor of *S. aureus*, its inhibition might be an important tool in treating *S. aureus* infections^59^. In contrast to the effect of pure silver ions and to the observations by Wang et al.^21^, expression of *ahpD* was reduced upon AgAu NP treatment, despite ROS activity being increased. It could thus not be concluded that AgAu NPs likewise impair the alkylhydroperoxidase D of the stress response system, but instead possibly promotes its stability by targeting feedback loops. Alternatively, the effect could be explained by the magnitude of antibacterial activity. Since AgAu NPs release a small amount of their silver into the solution as ions but AgNO_3_ dissolves completely, the latter cause a stronger antibacterial effect (Supplementary Fig. 1). Perhaps there are different damage thresholds at which the cells focus on specific pathways or different amounts of silver ions are required to inhibit different components of the cell. ROS activity also built up over time, so it might take some more time for gene expression to react. However, the exact mechanism would need to be clarified in future studies. In contrast, the biofilm inhibitor gene *icaR* was downregulated upon both, AgAu NP and silver ion treatment. This is in line with findings from Wang et al., who described a final downregulation of *icaR* at inhibitory silver concentrations^60^. This could again indicate a potential counterbalancing due to a direct or indirect promotion of the IcaR protein by silver. However, if silver indeed has the potential to impair *S. aureus* biofilm formation on the molecular level needs to be addressed in further studies. In this study, first insights could be achieved that the effect of AgAu NPs on *S. aureus* metabolic activity is based on targeting central molecular pathways like glycolysis with similarity to silver ions, which strengthens silver ions to be the major antibacterial agent of these nanoparticles.

## Conclusion

On the way towards alternative antibacterial substances to combat the increasing number of antibiotic-resistant bacterial strains, ultrapure, surfactant-free AgAu NPs with a gold molar fraction of 0.2 synthesized by laser ablation in liquids were analyzed in this study on different stages of *S. aureus* biofilm formation. Based on the selected experimental conditions of this study, it could be shown that AgAu NPs exhibit antibacterial properties against planktonic bacteria but also against early-stage and even mature biofilms, with a complete diffusion of the antibacterial species (NP or silver ions) through the biofilm matrix. Furthermore, AgAu NPs primarily targeted metabolic activity and to a smaller extend membrane integrity. This is in line with the known mechanism of pure silver ions, which was further supported by initial analysis on the molecular level. On this basis, future research should be conducted analyzing the underlying mechanisms in more detail and also aiming for further modifications that allow either for a more targeted antibacterial effect or an entire biofilm eradication based on the here studied promising silver–gold-alloy nanoparticles. A possible approach could be to include an inert support vessel, for example SiO_2_ or TiO_2_, to improve dispersion and prevent agglomeration. Testing the antibacterial activity against different also Gram-negative strains, for example the common pathogen *Pseudomonas aeruginosa*, should also be considered in future.

## Materials and methods

### Silver–gold nanoparticle synthesis

For all experiments, surfactant free colloidal AgAu NPs consisting of 80 mol% silver and 20 mol% gold were used. They were synthesized by pulsed laser ablation in liquids, using a 1064 nm Nd-YAG laser with 10 ns pulse length, 10 kHz repetition rate and a fluence of 23.5 J/cm^2^. For this purpose, pure solid metal targets of the respective alloy (0.5 mm thickness, 99.99% purity, Institute for Noble Metal and Metal Chemistry, Schwäbisch-Gmünd, Germany) were fixed in a self-constructed 30 mL PTFE batch chamber^61^. Size-quenching and electrostatic stabilization was achieved by adding a diluted electrolyte (0.2 mM NaCl) in Milli-Q water (18 MΩ resistance)^31,62,63^. During the synthesis, the liquid was continuously stirred to minimize shielding and reirradiation effects. A galvanometric scanner system (SCANcube10, SCANLAB GmbH, Puchheim, Germany) guided the pulses to an f-theta lens (f = 100 mm) which focused the pulses in a spiral pattern on the surface of the target. The ablations were carried out for 10 min each time. Nanoparticles were stored in the dark.

### Nanoparticle characterization

Colloidal nanoparticles were characterized by UV–Vis spectroscopy, transmission electron microscopy (TEM), and X-ray-fluorescence (XRF) spectroscopy. UV–Vis spectroscopy was done using a Thermo Scientific Evolution 201 spectrometer (Thermo Fisher Scientific Inc., Waltham, MA, USA) in a wavelength range 300–900 nm in a quartz cuvette with a path length of 10 mm. For TEM analysis, colloidal suspensions were drop-casted on a carbon-coated copper grid and analyzed using a Zeiss EM 910 instrument (Carl Zeiss Microscopy Deutschland GmbH, Oberkochen, Germany) with an acceleration voltage of 120 kV. The bulk composition of the colloid was measured via XRF using the Bruker WD XRF S8 Tiger (Bruker, Billerica, MA, USA). The standard QuantExpress method^64,65^ was used to analyze the colloid. Measurements were performed at 4 kW with a 34 mm aperture in a cuvette with a 4 µm Prolene® membrane from Chemplex Industries Inc. (Palm City, FL, USA). 9 g of the colloid sample was placed in the cuvette and measured in a 300 mbar helium atmosphere. AgAu NPs were deployed as surface concentrations based on procedures described elsewhere in more detail^33,38^. In short, the total surface area was determined from known mass concentrations of the colloids in combination with surface-weighted particle size distributions obtained either from TEM or analytical disk centrifugation, assuming spherical shape for the corresponding NPs.

### Bacterial strain and culture conditions

*Staphylococcus aureus* DSM 20,231 was obtained from the German Collection of Microorganisms and Cell Cultures GmbH (DSMZ, Braunschweig, Germany) and stored at − 80 °C as glycerol stocks. The bacteria were cultured overnight in tryptone soya broth (TSB, Oxoid Deutschland GmbH, Wesel, Germany) supplemented with 10% yeast extract (Carl Roth GmbH + Co. KG, Karlsruhe, Germany) (TSBy) at 37 °C under aerobic conditions and agitated at 200 rpm.

### Incubation of planktonic *S. aureus* with nanoparticles

After incubation overnight, the optical densities (OD_600_) of the bacterial cultures were measured (BioPhotometer, Eppendorf SE, Hamburg, Germany) and adjusted to 1. Three biological replicates were used. 100 µL of bacteria suspension were mixed with 900 µL of 0.2 mM NaCl (Sigma Aldrich, St. Louis, MO, USA) or binding buffer (100 mM NaCl, 20 mM Tris, 10 mM MgCl_2_, 5 mM KCl, 1 mM CaCl_2_, 0.005% Tween 20, all Carl Roth GmbH + Co. KG, pH 7.6)^41^ containing different surface concentrations of AgAu NPs, for a final OD_600_ of 0.1. No precipitation upon dilution of nanoparticles was observed. The chosen surface concentrations were 0.625 cm^2^/mL, 1.25 cm^2^/mL, 2.5 cm^2^/mL, and 5 cm^2^/mL. 200 µL of each mixture were then added to a 96-well plate (Thermo Fisher Scientific Inc.) with four technical replicates for each biological replicate and incubated in the dark for 3 h at 37 °C under aerobic conditions and agitation at 450 rpm.

### Incubation of *S. aureus* biofilms with nanoparticles

For biofilm formation, overnight cultures were diluted to an OD_600_ of 0.1 in TSBy supplemented with 50 mM glucose (Carl Roth GmbH + Co. KG) and subsequently applied to either 6-well plates (for microscopic analysis; Greiner Bio-One GmbH, Frickenhausen, Germany) or 96-well plates (for analysis of metabolic activity) and incubated in the dark for 5 h and 24 h at 37 °C under aerobic conditions without agitation, respectively. Formed biofilms were washed once with 0.2 mM NaCl or binding buffer and then incubated with different concentrations of AgAu NPs in 0.2 mM NaCl or binding buffer for 18 h at 37 °C under aerobic conditions and agitation at 300 rpm.

### Quantifying bacterial metabolic activity by resazurin assay

To analyze viability of *S. aureus* after incubation with AgAu NPs, the metabolic activity was measured using an established resazurin assay^44^ with a slight modification. For planktonic bacteria, 30 µL of 0.01% resazurin (Sigma Aldrich) in 0.2 mM NaCl or binding buffer mixed with 10% TSBy were added to each well. For biofilms, wells were washed once with 0.2 mM NaCl or binding buffer and afterwards 100 µL of 0.001% resazurin in 0.2 mM NaCl or binding buffer containing 10% TSBy supplemented with 50 mM glucose were added. The mixtures were then incubated in the dark for 90 min at 37 °C under aerobic conditions and agitation at 450 rpm. Afterwards, fluorescence signal was measured using a plate reader (M200 PRO, Tecan Group AG, Männedorf, Switzerland) with an excitation wavelength of 530 nm and an emission wavelength of 590 nm.

### Biofilm fluorescence staining, confocal laser scanning microscopy and software-based image analysis

Biofilm volume and membrane integrity were quantified by staining biofilms with the fluorescent dyes SYTO®9 and propidium iodide using the LIVE/DEAD® BacLight™ Bacterial Viability Kit (Life Technologies, Darmstadt, Germany) and acquiring images by confocal laser scanning microscopy (CLSM, Leica SP-8, Leica Microsystems, Wetzlar, Germany). The dye stock solutions were applied to biofilms in a concentration of 1:2000 in phosphate buffered saline (PBS, Sigma Aldrich) for 15 min. Following staining, biofilms were fixated by incubating them for 15 min at 4 °C in 2.5% glutardialdehyde (Carl Roth GmbH + Co. KG) in PBS. For microscopy, biofilms were immersed in PBS and a 63 × water immersion objective was used. Total magnification was 630x. Biofilms were excited using 488 nm and 552 nm lasers. Emission was detected at 500–540 nm for SYTO®9 (green), which stains all cells, and at 675–750 nm for propidium iodide (red), which only stains damaged cells and is supposed to replace SYTO®9. Consequently, cells stained by both dyes were defined as damaged cells. Image stacks were acquired in a 1024 × 1024 pixel area with a z-step size of 2 µm. Image data processing was done using the Imaris × 64 8.4.1 software package (BitPlane AG, Zurich, Switzerland), where volumes of green and red channels were evaluated using the Surface function. Volume containing both dyes was afterwards subtracted from the green volume by colocalization analysis.

Furthermore, single z-planes within biofilms were evaluated for membrane integrity to analyze diffusion of NPs through biofilms. Leica Application Suite (LAS X, Leica Microsystems) was used to extract single images from the data and ImageJ 1.48v (Wayne Rasband, National Institutes of Health, Bethesda, MD, USA) was used to analyze the images. Planes were taken starting from the bottom of the biofilm and subsequently every 4 µm.

### RNA isolation, cDNA synthesis and qRT-PCR

To analyze the effect of AgAu NPs on *S. aureus* on the transcription level, 1 mL of *S. aureus* overnight culture was added to 19 mL of fresh TSBy and incubated for 3 h. Afterwards, the OD_600_ was measured and adjusted to 0.1 in 20 mL of binding buffer, either with or without 1.25 cm^2^/mL AgAu NPs. This mixture was then incubated in the dark for 3 h at 37 °C under aerobic conditions and agitation at 200 rpm. After centrifugation at 4000 g for 15 min, the resulting pellet was rinsed with PBS once and centrifugation was repeated. The pellet was diluted in RNAprotect (QIAGEN GmbH, Hilden, Germany) and incubated for 5 min at room temperature as 750 µL aliquots. Afterwards, bacteria were harvested by centrifugation at 5000 g for 10 min and stored at − 20 °C until RNA isolation. This entire process was repeated with 1 µg/mL dissolved AgNO_3_ instead of AgAu NPs for a comparison with silver ions.

For RNA isolation, the QIAGEN® RNeasy® Mini Kit (QIAGEN GmbH) was used as described in the manufacturer’s protocol. The isolated RNA was additionally treated with RNase-Free DNase Set (QIAGEN GmbH) to remove genomic DNA from the samples. To quantify the amount of RNA and determine its quality, samples were measured using a 2100 Bioanalyzer system (Agilent Technologies, Santa Clara, CA, USA). Afterwards, RNA was transcribed to cDNA using the QuantiTect® Reverse Transcription Kit (QIAGEN GmbH) according to the manufacturer’s protocol. For quantification of gene expression, quantitative real-time polymerase chain reaction (qRT-PCR) was performed using the iQ™ SYBR® Green Supermix (Bio-Rad Laboratories GmbH, Feldkirchen, Germany) following the manufacturer’s protocol. As potentially influenced genes, *pfkA* (ATP-dependent 6-phosphofructokinase), *ahpD* (Alkylhydroperoxidase)*,* and *icaR* (Biofilm operon *icaADBC* HTH-type negative transcriptional regulator) were chosen and *rrsA* (ribosomal RNA^66^) was chosen as housekeeping gene. Primers, temperatures, elongation times, and number of cycles are given in Supplementary Table 3 and Supplementary Table 4. The qRT-PCR data were analyzed using the 2^−ΔΔ*C*^_T_-method^39^.

### Total ROS activity

To analyze if AgAu NPs induced ROS activity, the Cell Meter™ Fluorimetric Intracellular Total ROS Activity Assay Kit*Green Fluorescence* (AAT Bioquest, Inc., Pleasanton, CA, USA) was used according to the manufacturer’s protocol. Overnight cultures of *S. aureus* were diluted to an OD_600_ of 2.2 in binding buffer and 100 µL per well were added to a 96-well plate. 100 µL of Amplite™ ROS Green working solution per well was then added to the bacterial suspension. The mixture was then incubated in the dark for 1 h at 37 °C. Afterwards, AgAu NPs were diluted to a surface concentration of 13.75 cm^2^/mL in binding buffer and 20 µL per well were added to the well plate for a final concentration of 1.25 cm^2^/mL. The well plate was then incubated in the dark at 37 °C and fluorescence measurements were taken using a plate reader with an excitation wavelength of 490 nm and an emission wavelength of 525 nm after 30 min, 1 h, 2 h, and 3 h. Untreated bacteria were used as a control. Data was normalized to the control for each time point.

### Statistical analysis

Data presentation and statistical analysis were done using GraphPad Prism 8.4 (GraphPad Software Inc., San Diego, CA, USA). Every set of data was tested for normal distribution and consequently analyzed for statistical significance. If data were normally distributed, it was analyzed by one-way ANOVA or unpaired t tests, otherwise it was analyzed by Kruskal Wallis’ or Friedman’s test, each with a multiple comparisons test. The threshold for statistical significance for all comparisons was defined with α = 0.05.

### Supplementary Information

Supplementary Information.

## Data Availability

The datasets used and/or analyzed during the current study are available from the corresponding authors (heine.nils@mh-hannover.de or stiesch.meike@mh-hannover.de) on reasonable request.
