# Supplementary material for: Anti-biofilm properties of laser-synthesized, ultrapure silver–gold-alloy nanoparticles against Staphylococcus aureus
Source: Sci Rep. 2024 Feb 10;14:3405. doi: 10.1038/s41598-024-53782-x (PMC10858226; doi:10.1038/s41598-024-53782-x)
Supplement: Supplementary file 1 — Supplementary Information. [file 41598_2024_53782_MOESM1_ESM.docx]

**Supplementary Information for:**

**Anti-Biofilm Properties of Laser-Synthesized, Ultrapure Silver-Gold-Alloy Nanoparticles against *Staphylococcus aureus***

Nils Heine^1,2^*, Katharina Doll-Nikutta^1,2^, Frederic Stein^3^, Jurij Jakobi^3^, Alexandra Ingendoh-Tsakmakidis^1,2^, Christoph Rehbock^3^, Andreas Winkel^1,2^, Stephan Barcikowski^3^, Meike Stiesch^1,2^*

^1^Department of Prosthetic Dentistry and Biomedical Materials Science, Hannover Medical School, Carl-Neuberg-Straße 1, 30625 Hannover, Germany

^2^Lower Saxony Centre of Biomedical Engineering, Implant Research and Development, Stadtfelddamm 34, 30625 Hannover, Germany

^3^Technical Chemistry I, University of Duisburg Essen, Universitaetsstr. 7, 45141 Essen, Germany

*Corresponding author: [meike.stiesch@mh-hannover.de](mailto:meike.stiesch@mh-hannover.de), [heine.nils@mh-hannover.de](mailto:heine.nils@mh-hannover.de)

|  | ^0 cm²/mL^ | ^0.625 cm²/mL^ | ^1.25 cm²/mL^ | ^2.5 cm²/mL^ | ^5 cm²/mL^ |
| --- | --- | --- | --- | --- | --- |
| ^0 cm²/mL^ | ^-^ | ^*^ | ^*^ | ^*^ | ^*^ |
| ^0 .625 cm²/mL^ | ^*^ | ^-^ | ^*^ | ^*^ | ^*^ |
| ^1.25 cm²/mL^ | ^*^ | ^*^ | ^-^ | ^*^ | ^*^ |
| ^2.5 cm²/mL^ | ^*^ | ^*^ | ^*^ | ^-^ | ^-^ |
| ^5 cm²/mL^ | ^*^ | ^*^ | ^*^ | ^-^ | ^-^ |

Supplementary Table 1: Significance table for NaCl in Figure 2. Statistically significant differences with p ≤ 0.05 determined by an ordinary one-way ANOVA with Tukey’s test for multiple comparisons are indicated by (*).

|  | ^0 cm²/mL^ | ^0.625 cm²/mL^ | ^1.25 cm²/mL^ | ^2.5 cm²/mL^ | ^5 cm²/mL^ |
| --- | --- | --- | --- | --- | --- |
| ^0 cm²/mL^ | ^-^ | ^*^ | ^*^ | ^*^ | ^*^ |
| ^0 .625 cm²/mL^ | ^*^ | ^-^ | ^*^ | ^*^ | ^*^ |
| ^1.25 cm²/mL^ | ^*^ | ^*^ | ^-^ | ^*^ | ^*^ |
| ^2.5 cm²/mL^ | ^*^ | ^*^ | ^*^ | ^-^ | ^-^ |
| ^5 cm²/mL^ | ^*^ | ^*^ | ^*^ | ^-^ | ^-^ |

Supplementary Table 2: Significance table for binding buffer in Figure 2. Statistically significant differences with p ≤ 0.05 determined by an ordinary one-way ANOVA with Tukey’s test for multiple comparisons are indicated by (*).


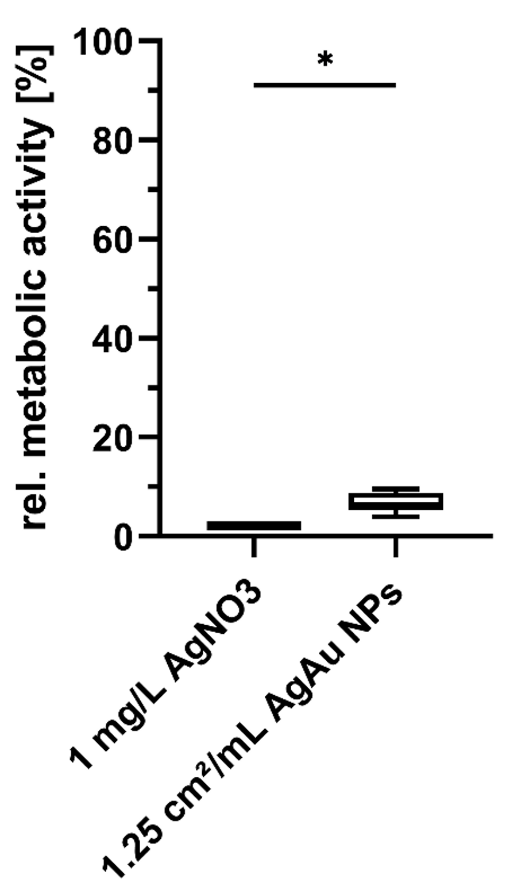


Supplementary Figure 1: Relative metabolic activity of planktonic S. aureus treated with AgNO_3_ or nanoparticles consisting of 80 mol% silver and 20 mol% gold in binding buffer. Statistically significant difference with p ≤ 0.05 is indicated by (*) and was determined by an unpaired t test.

| Primer | Sequence (5’ to 3’) |
| --- | --- |
| *rrsA* forward | GCCCCTTAGTGCTGCAGCTA |
| *rrsA* reverse | AGTTTCAACCTTGCGGTCGTA |
| *pfkA* forward | GTCCAGAGTTTAAGGAGCAAGA |
| *pfkA* reverse | ACTACCGTCACCACCAATAAC |
| *ahpD* forward | GTCTGTAATGAGCCAAGGTGAA |
| *ahpD* reverse | GGATATCAACACAATAATGACAACCG |
| *icaR* forward | TCGAACTATTCAATTGATGCTTTA |
| *icaR* reverse | CAGAAAATTCCTCAGGCGTA |

Supplementary Table 3: Primers for qRT-PCR.

| Gene | Temperature [°C] | Elongation Time [s] | Number of cycles |
| --- | --- | --- | --- |
| *rrsA* | 60 | 30 | 40 |
| *pfkA* | 60 | 30 | 40 |
| *ahpD* | 63 | 30 | 40 |
| *icaR* | 55 | 30 | 40 |

Supplementary Table 4: qRT-PCR annealing temperatures, elongation times, and number of cycles for each gene.
